# Do we see what we should see? Describing non-covalent interactions in protein structures including precision

**DOI:** 10.1107/S2052252513031485

**Published:** 2013-12-05

**Authors:** Manickam Gurusaran, Mani Shankar, Raju Nagarajan, John R. Helliwell, Kanagaraj Sekar

**Affiliations:** a Supercomputer Education and Research Centre, Indian Institute of Science, Bangalore, Karnataka 560 012, India; b School of Chemistry, University of Manchester, Brunswick Street, Manchester M13 9PL, England

**Keywords:** salt bridges, crystal structure analysis, protein crystal structure precision, full-matrix least-squares method, measurement uncertainty, diffraction-component precision index, DPI, neutron diffraction

## Abstract

The power of X-ray crystal structure analysis as a technique is to ‘see where the atoms are’. The results are extensively used by a wide variety of research communities. However, this ‘seeing where the atoms are’ can give a false sense of security unless the precision of the placement of the atoms has been taken into account. Indeed, the presentation of bond distances and angles to a false precision (*i.e.* to too many decimal places) is commonplace. This article has three themes. Firstly, a basis for a proper representation of protein crystal structure results is detailed and demonstrated with respect to analyses of Protein Data Bank entries. The basis for establishing the precision of placement of each atom in a protein crystal structure is non-trivial. Secondly, a knowledge base harnessing such a descriptor of precision is presented. It is applied here to the case of salt bridges, *i.e.* ion pairs, in protein structures; this is the most fundamental place to start with such structure-precision representations since salt bridges are one of the tenets of protein structure stability. Ion pairs also play a central role in protein oligomerization, molecular recognition of ligands and substrates, allosteric regulation, domain motion and α-helix capping. A new knowledge base, SBPS (Salt Bridges in Protein Structures), takes these structural precisions into account and is the first of its kind. The third theme of the article is to indicate natural extensions of the need for such a description of precision, such as those involving metalloproteins and the determination of the protonation states of ionizable amino acids. Overall, it is also noted that this work and these examples are also relevant to protein three-dimensional structure molecular graphics software.

## Abstract 2

The presentation of non-covalent interactions in protein X-ray crystal structures needs to routinely include their atomic precision, as detailed here; a user knowledge base for these precisions with examples is also offered. Cases are also indicated where the need for such a description of precision is a natural extension, such as those involving metalloproteins and the protonation states of ionisable amino acids. This study is also relevant to protein three-dimensional structure molecular-graphics software.

## 1. Introduction   

The intricate three-dimensional structures of biomolecules describe their functionalities, which can be determined accurately by the power of X-ray crystallography. X-ray crystal structures are in fact a space and time average in which the atoms in a crystal do not exist fixed within a rigid body, *i.e.* with fixed three-dimensional coordinates, but exhibit an independent motion about their centre of mass over time and also a spatial average of all of the molecular copies over the crystal lattice. To understand the various allosteric regulation, catalytic and interatomic interactions, a static crystal structure, even with its atomic displacement parameters, is not adequate as a descriptor. The diffraction precision index (DPI; Cruickshank, 1999 ▶; Blow, 2002 ▶), derived from experimental crystallographic parameters, is now available for use to estimate the experimental precision of the atomic coordinates in a protein structure over a much wider range of diffraction resolutions and in effect for all cases. To this end, we advocate this straightforward method of properly estimating the precision of atomic placement. This can be derived from the overall experimental diffraction conditions and the measured data quality, as well as the standard reliability-factor statistics of the finalized protein model refinement (*i.e.* the *R* factors in the equations for the DPI; see below). Finally, we harness the atomic displacement parameters of any individual pair of interacting atoms and we can thus quantify the precision of any given interatomic distance. Thereby, the presentation of protein crystal structures needs to routinely include their atomic precision, which we detail over all current Protein Data Bank (PDB; Berman *et al.*, 2000 ▶; http://www.rcsb.org) entries; we also offer a user knowledge base with structural biology examples.

Biological X-ray crystallographic structure determination (‘macromolecular crystallography’) is conducted at a wide variety of diffraction resolutions. Some protein crystal structures reach a diffraction precision akin to that of chemical crystallography (Deacon *et al.*, 1997 ▶), and in such studies directly calculated atomic coordinates and atomic displacement parameters (ADPs) along with their associated estimated errors (‘standard uncertainties’) are immediately available, for example *via* the ‘*SHELX*’ chemical crystallo­graphy style of model refinement [see Sheldrick (2008 ▶) for a summary and Deacon *et al.* (1997 ▶) and Ahmed *et al.* (2007 ▶) for examples of such protein refinement]. At poorer diffraction resolutions such full-matrix inversion methods are not possible owing to an insufficient X-ray diffraction data to molecular model parameter ratio; Ahmed *et al.* (2007 ▶) scrutinized the diffraction resolution at which full-matrix inversion ‘breaks down’, which was around 1.5 Å. As explored by Cruickshank (1999 ▶), the agreement of the DPI with full-matrix error estimates, where they can be calculated, is very good. Our new knowledge base, SBPS (Salt Bridges in Protein Structures), is the first to take these DPI-derived structural precisions into account.

Salt bridges (ion pairs) are the interactions between the side-chain atoms (nitrogen and oxygen) of oppositely charged amino-acid residues; the formal analogy of the term is derived from sodium and chloride ion interactions, and hence the term used is ‘salt bridge’. They are are one of the tenets of protein structure stability, and also play a central role in protein oligomerization, molecular recognition of ligands and substrates, allosteric regulation, domain motion, and α-helix capping (Perutz, 1970 ▶; Fersht, 1972 ▶; Barlow & Thornton, 1983 ▶; Musafia *et al.*, 1995 ▶; Xu *et al.*, 1997 ▶; Kumar *et al.*, 2000 ▶; Kumar & Nussinov, 2002 ▶). The side-chain N atoms of the basic residues arginine (NH1 and NH2), histidine (N^δ1^ and N^∊2^) and lysine (N^ζ^) and the side-chain O atoms of the acidic residues aspartate (O^δ1^ and O^δ2^) and glutamate (O^∊1^ and O^∊2^) participate in such ion-pair formation (Gowri Shankar *et al.*, 2007 ▶). We illustrate our approach of using the DPI to derive ion-pair bond-distance error estimates with the specific important biological examples of cortexillin and isoaspartyl dipeptidase, which are proteins that play fundamental roles in cytokinesis and the protein-degradation pathway, respectively. The DPI-based structural precision approach of our knowledge base can readily be extended to other cases such as the metalloprotein knowledge base MESPEUS, a database of the geometry of metal sites in proteins (Hsin *et al.*, 2008 ▶).

## 2. Materials and methods   

### 2.1. The Salt Bridges in Protein Structures (SBPS) database   

SBPS is a knowledge base created to provide information pertaining to the ion pairs present in all the three-dimensional protein structures available in the PDB archive. The knowledge base, in general, contains two main parts: (i) ion pairs and (ii) water-mediated ion pairs. For each part, users can perform a general search and an advanced, more detailed, search. Criteria such as the organism name, molecular classification, author, protein name and enzyme can be employed during both searches. In addition, the user can further narrow down a search to obtain specific queries based on the following criteria.

(i) The ion-pair type: whether it is a complete or an incomplete ion pair.
(ii) The structural environment of the ion pair: whether it is across protein subunits (inter-subunit) or within protein subunits (intra-subunit) and the amino-acid-residue specificity: interactions between the possible combinations of acidic and basic residues (Asp–His, Asp–Arg, Asp–Lys, Glu–His, Glu–Arg or Glu–Lys).

A *Jmol* graphical plug-in (http://www.jmol.org/) is incorporated in the SBPS database to allow users to visualize each ion pair along with its distance and standard deviation. The knowledge base is updated weekly and is freely accessible over the World Wide Web at http://cluster.physics.iisc.ernet.in/sbps.

### 2.2. Calculation of atomic position standard deviations   

Cruickshank introduced the DPI (Cruickshank, 1999 ▶) as a quantitative indicator of the average uncertainty of the position (*i.e.* the coordinate) of each of the atoms within a protein structure. As explained by Cruickshank (1999 ▶), it is a precision not a mathematical accuracy treatment as it does not attempt to cover systematic errors; fortunately, as synchrotron and home-laboratory X-ray source intensities and detector technology have improved, smaller samples are used, and X-ray absorption, which was a common systematic error before these developments, is not often remarked upon. Likewise, electronic area detectors have greatly improved the number of reflections used in unit-cell parameter estimates, now often with the calculation of standard uncertainties. However, in both cases systematic errors may remain in X-ray biological crystal structure analyses, certainly for historical PDB entries.

The coordinate error of each atom can be calculated using (1) and takes account of the *B* factor of an individual atom *versus* that of an average atom,
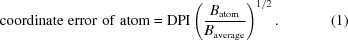
Obviously, the quality of an atomic position estimate from (1) depends explicitly on the quality of the determination of each of the *B* factors for the pair of atoms in question. This has historically been recognized as an area of difficulty, *i.e.* obtaining the correct atomic *B*-factor estimates, whether isotropically refined or anisotropically refined. However, there have been a range of technical improvements to the experiment and to the software for diffraction data processing and macromolecular model refinement. *B* factors are routinely quoted to a precision of one decimal place. That said, this may be inappropriate, especially for lower resolution refinements, where *B* factors may well be best quoted only to the nearest integer. These immediate comments give a guide as to how well the DPI-derived σ on an ion-pair distance may be vulnerable to such *B*-value errors.

Therefore, in displaying distances in molecular-graphics programs, explicit use of the distance error is possible for two atoms of interest within the PDB structure. It is also computationally inexpensive to calculate. Interacting atoms, such as ion pairs, a metal and its ligands, or hydrogen-bonded van der Waals attracted atom pairs, are ideal for such a DPI mathematical treatment of the error in the estimated separation distance. Obviously, two covalently bonded atoms are held *via* restraints to a chemical dictionary value. Cruickshank (1999 ▶) discussed covalent-bond geometry dictionary restraints. Restraints are needed even in atomic resolution macromolecular model refinement, notably for the more mobile portions, such as loops, which do not diffract to atomic resolution. Thus, their ADPs (‘*B* factors’) are higher than the average and this fuels a large increase in the DPI for these atoms. Any such ion-pair bond distance, the focus of this article, would thereby be relatively poorly determined and our use of the DPI here is a descriptor of that imprecision.

The DPI equation uses the protein atomic model refinement *R* (reliability) factor (either *R* or *R*_free_), the diffraction data completeness, the diffraction resolution, the overall (*i.e.* average) atom *B* factor and the diffraction data to parameter ratio as parameters in order to estimate the overall coordinate uncertainty of an ‘average atom’,
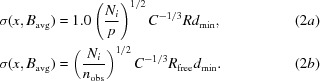



Cruickshank’s form of the DPI is shown in (2), where σ(*x*, *B*_avg_) is the DPI for an atom with an average *B* factor, *N*_*i*_ is the number of fully occupied atoms of type *i*, *p* = *n*_obs_ − *n*_params_, *C* is the completeness of the diffraction data, *R* is the *R* factor (or *R*_free_) and *d*_min_ is the diffraction resolution. In this form, it was difficult to assess how some of the parameters in the equation were related to the experimental parameters of the diffraction resolution or completeness, and therefore Blow (2002 ▶) later extended the derivation to relate them to parameters that are readily available to an experimentalist, 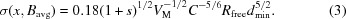
The Blow version of the DPI descriptor now explicitly depends on the solvent content *s* (= *N*_solv_/*N*_atoms_), the Matthews volume (of protein in the unit cell) *V*_M_ (Matthews, 1968 ▶), the diffraction data completeness, the *R*_free_ and the diffraction resolution. Direct comparisons against diffraction resolution and completeness (*e.g.* in attempts to make an improved diffraction data experiment) can now readily be made (Fisher *et al.*, 2008 ▶, 2012 ▶; Tanley *et al.*, 2012 ▶).

**Figure 1:**
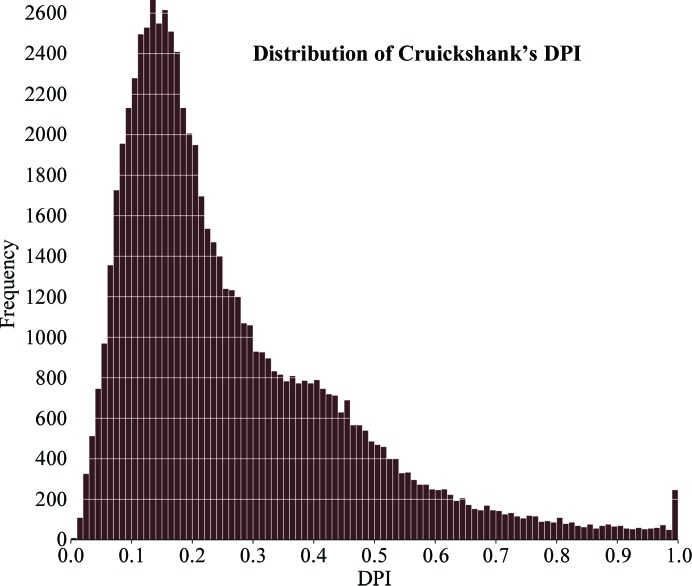
The DPI values calculated for the whole of the PDB (as of 20 August 2013) using (2) and plotted as a histogram of values. The most probable value is 0.15 Å, and thus the quite common display of distances and angles to two decimal places for non-bonded interactions such as ion pairs is an incorrect level of precision, from which misleading conclusions on structural chemistry will be made. Note that the histogram shows DPI values up to 1 Å and thus the appropriate representation of interaction distances in these cases should be only as integers and not real numbers with decimal places; thus, such cases, whether used for modelling or for protein-folding energetics derived from these coordinate files, must be treated with considerable caution.

In Fig. 1 ▶, using (2), we show a histogram of the DPI values for (nearly) all of the PDB X-ray crystal structure entries (described below). The coordinate error of each atom can be calculated using (1) and takes account of the *B* factor of an individual atom *versus* that of an average atom.

Therefore, explicit use of the bond-distance error is possible in displaying distances in molecular-graphics programs *via* (1) and (2) for a given PDB entry. However, molecular-graphics programs to our knowledge commonly show bond distances within any structure to two decimal places irrespective of their diffraction resolution! Likewise, the considerable variation in the *B* factors of atoms forming different chemical bonds is not recognized either in such protein structure representations. Users of the PDB have a wide range of science backgrounds, which are very often not crystallo­graphic. Thus, we see such representations of precision in a protein structure as a vital development as a firm guide to users of protein structure coordinates.

This use of the DPI could be extended to cases of macromolecular model refinement using X-ray and neutron diffraction data, in which the number of observations and parameters are increased accordingly in the DPI formulae that we have harnessed above. However, the specific case of heavy-water-soaked crystals, as opposed to deuterium-based microbiological protein expression, is more complicated because the unexchangeable H atoms, *e.g.* those on C atoms, have negative nuclear scattering factors. In any case, we would comment that the usual use of neutron diffraction is to improve the *accuracy* of the chemical knowledge of the protonation state of ionizable amino-acid side chains rather than the atomic coordinate precision. An interesting case of ensuring both chemical accuracy and coordinate precision is that of the use of neutron diffraction to determine the chemical state of key bound waters (as neutral molecules or hydroxyl or hydronium ions) *and* their geometric orientation.

### 2.3. DPI values calculated for the whole of the PDB   

The DPI was calculated using (2*a*) or (2*b*) according to Cruickshank’s criterion of where *p* becomes negative in (2*a*), thus making the DPI imaginary for low-resolution structures, as the number of parameters may exceed the number of diffraction data; therefore, as an expedient, (2*b*) was validated by Cruickshank for use instead. As of 20 August 2013, there were 82 435 crystal structures in the PDB. Several ‘screening filters’ were used. Firstly, each PDB entry had to have the relevant parameters provided by the depositor in order to be able to use Cruickshank’s equations. Secondly, we suggest that it is reasonable that the overall completeness of a diffraction data set be >75%. Thirdly, it is required (as per Cruickshank’s theory) that the percentage of fully occupied atoms is by far the dominant character of a given PDB structure, and we suggest that this needs to be >90%. Thus, overall, a DPI was not calculated for ∼10% of the available structures.

Finally, forming what might be called a problematic cohort of structures, there are 618 PDB structures whose DPI values are greater than 2 Å. Detailed analyses reveal very obvious reasons for this. Some are very low resolution crystal structures; these include depositions, for example, from the very early use of X-ray lasers, where the geometric detector coverage extended to only 8 Å, but also other cases where high-resolution protein subunits have been rigid-body-refined into place with low-resolution data, *e.g.* in a new crystal form of a complex involving these protein subunits. We removed the use of the DPI from these structures in our knowledge base for the simple reason that detailed analyses of non-covalent inter­actions is not appropriate. However, there are occasional PDB codes at ostensibly good diffraction resolution, *e.g.* 2.0 Å, where the DPI value is much higher than those of crystal structures with an otherwise identical resolution limit. Within the cohort of approximately 72 000 structures that survived the above filters, there are 2272 PDB structures whose DPI values are greater than or equal to 1 Å. Within the knowledge base we make an admittedly somewhat arbitrary filter that for any PDB entry with a DPI of >1 Å a message is given to the user that ‘the DPI for this PDB code is so large (>1 Å) as to make a precision estimate of any non-bonded distance to be of too low a confidence to be usable in this way’.

### 2.4. SBPS database implementation and access   

The SBPS knowledge base was developed with Perl/CGI and Perl/DBI modules. In SBPS, the data are implemented in MySQL under the stable operating system Solaris 10 (Intel Xeon Quad core 2.66 GHz, 4 GB FDIMM main memory). This operating system was particularly chosen for its security, scalability and reliability. The computing resource has been tested using various platforms (Mac OS, Windows, Linux and Solaris) with all reliable web browsers. The knowledge base has been thoroughly validated and in general is very fast. However, the response time may vary depending upon the network speed and the number of users accessing the knowledge base at a given time.

The user can access the knowledge base in two ways: (i) ‘Search set’ and (ii) ‘Explore’. The user can assess the entire knowledge base, based on the criteria of ion pairs (described in detail below), by using the ‘Search Set’ option. Using the ‘Explore’ option, the user can study the ion pairs present in a single PDB file.

#### 2.4.1. Some further runtime details   

Users can view the detailed results by clicking on the corresponding PDB code from the table or can save the results in the form of a PDF file by clicking on the ‘PDF’ option provided. In the detailed results page, options are provided (on the left side of the web page) for dynamically choosing specific acidic and basic residue combinations along with other options for selecting interactions occurring across subunits (Inter) and within subunits (Intra). This option could be used to analyse the occurrence of ion pairs in particular chain(s) and/or between chain(s). In addition, the ‘Parameter’ option can be used to select ion pairs based on distance and angle (exclusively for water-mediated ion pairs) criteria.

After query submission, the user obtains a results table containing a list of PDB codes along with detailed information on the number of ion pairs occurring in each combination (residue specificity) and their location (inter and intra).

The option ‘Complete/Incomplete salt bridges’, found below the results table, can be used to visualize the complete and incomplete ion pairs present in a particular PDB entry. Secondly, the option ‘Secondary structure’ facilitates the users with the residues of the ion-pair interaction that are involved in the formation of different secondary structures. Here again the user can dynamically sort through the three categories (α-­helix, β-sheet and coil). The results page displays detailed information on the donor and acceptor residues along with their atom and chain information and ion-pair distances with a calculated standard deviation.

## 3. Case studies   

### 3.1. Role of ion pairs in cortexillin   

**Figure 2:**
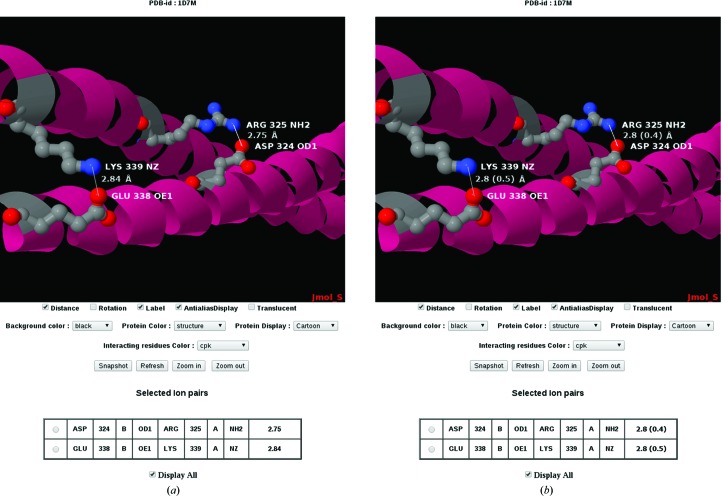
The protein structure of cortexillin I (PDB entry 1d7m) contains 19 inter-chain salt bridges that hold the dimer together [only two ion pairs (Asp324 O^δ1^ *B*–Arg325 NH2 *A* and Glu338 O^∊1^ *B*–Lys339 Nz *A*) are shown for clarity]. (*a*) Displayed without σs to demonstrate how misleading this is to the unwary reader or user and (*b*) displayed with σs and an appropriate number of decimal places.

*Dictyostelium discoideum* (slime mould) has been progressively used as a model organism for human disease analysis. In *D. discoideum*, cortexillin I and II are actin-bundling proteins that play a fundamental role in cytokinesis and are required to maintain a normal cleavage furrow during mitotic cell division. Mutational studies on this organism indicated that a dearth of cortexillins leads to centrosome amplification and to spindle abnormality (Williams *et al.*, 2006 ▶; Effler *et al.*, 2006 ▶). To analyse the role of ion pairs in cortexillin structures, we used our SBPS protein structure database to select cortexillin I crystal structures determined with a diffraction resolution of between 2 and 3 Å and with a quality (*i.e.* *R* factor) of between 10 and 30%. As an example, PDB entry 1d7m (Burkhard *et al.*, 2000 ▶) with a diffraction resolution of 2.7 Å, an *R* of 20.5% and an *R*_free_ of 24.9% is selected here. There are a total of 35 ion pairs for cortexillin I. These can be studied in specific categories using our database: 19 ion pairs are across the subunits and 16 ion pairs are within the subunits. It is interesting to note that of the 35 ion pairs, a total of two inter-chain salt bridges are found between glutamate/lysine and aspartate/arginine (Fig. 2 ▶) which hold the dimer together. Using the ‘Secondary structure’ option of SBPS, it can be seen that all of the ion-pair interactions occur within or between the helical regions of the protein structure. Upon further analysis (results not shown), it can be observed that the intrahelical ion pairs are crucial for the stability of monomeric α-helices and that the interhelical ion pairs are essential for their proper alignment and for coiled-coil orientation. A precise energy-based calculation of the contribution of the ion pairs to protein stability would ordinarily use these distances, but a proper account can now also be taken of their structural precision.

### 3.2. Role of water-mediated ion pairs in isoaspartyl dipeptidase   

**Figure 3:**
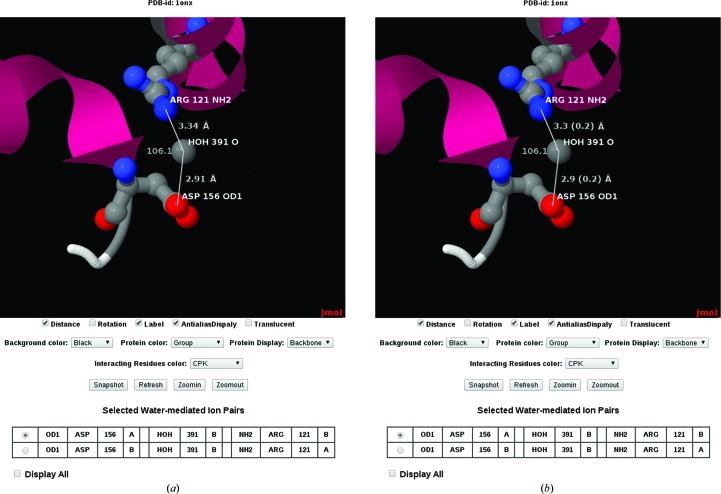
Two water-mediated ion pairs between aspartate and arginine residues (Asp156 O^δ1^ *A*–HOH391 O–Arg121 NH2 *B* and Asp156 O^δ1^ *B*–HOH391 O–Arg121 NH2 *A*) of different subunits in PDB entry 1onx. (*a*) Displayed without σs and (*b*) displayed with σs and an appropriate number of decimal places.

*Escherichia coli* is one of the well studied model organisms among prokaryotes. In *E. coli*, the enzyme isoaspartyl dipeptidase (IAD) is a member of the amidohydrolase protein superfamily and plays a major role in the protein-degradation pathway. It catalyses the hydrolytic cleavage of β-l-isoaspartyl linkages in a dipeptide and prevents their accumulation in the cell after protein degradation (Thoden *et al.*, 2003 ▶). To analyse the role of water-mediated ion pairs in IAD, the terms ‘*Escherichia coli*’, ‘Hydrolase’ and ‘Isoaspartyl Dipeptidase’ are given as input to the advanced search option under ‘Organism’, ‘Molecular classification’ and ‘Macromolecule name’, respectively. Based on the query and parameters such as resolution (between 1 and 3 Å), *R* factor (between 10 and 20%) and ‘greater than or equal to 2’ for the number of water-mediated ion pairs, six protein structures (PDB entries 1onw, 1onx, 1po9, 1ybq, 2aqo and 2aqv) are retrieved. From the results table, PDB entry 1onx (Thoden *et al.*, 2003 ▶) is selected here (high-resolution X-ray structure of iso­aspartyl dipeptidase from *E. coli*) determined at 2.1 Å resolution with *R* = 18.2% and *R*_free_ = 24.6%. There are a total of ten water-mediated ion pairs. Of these ten, eight water-mediated ion pairs occur within the subunits and the remaining two occur across the subunits (distance cutoff value of 2.5–3.5 Å; Fig. 3 ▶). By analysing the two interactions between subunits *A* and *B*, it can be observed that the bond angles exceeded 100°. This particular phenomenon implies that two oppositely charged residues separated by a large distance (>3.5 Å) require the aid of a water molecule to mediate the ion-pair interaction. Furthermore, with the help of the ‘Secondary structure’ option, it was found that the interactions mentioned above occur between an α-helix and a coil. It could be inferred that the hydration of such ion pairs plays a vital role in the stabil­ization of the dimeric structural conformations.

### 3.3. Metalloproteins and a properly quantitative biological inorganic chemistry   

There is currently just one place that metalloproteins can be shown: MESPEUS (http://mespeus.bch.ed.ac.uk/MESPEUS/). Supporting Fig. S1 shows a screenshot of the MESPEUS view of the Mn site in concanavalin A (PDB entry 1nls; Deacon *et al.*, 1997 ▶), *i.e.* the associated PDB file. Notice that not only are the σs not displayed by MESPEUS, but also, and worse, the Mn—O19 distance, for example, has been wrongfully truncated to two decimal places! (The σ from full-matrix inversion is 0.005 Å.) Therefore, a future enhancement of the otherwise excellent capabilities of MESPEUS would be to show the metal-to-ligand distances with their σs displayed. These can be either from full-matrix estimates or, if need be, as will usually be the case, based on the DPI.

## 4. Discussion   

### 4.1. Areas of application   

This is the first knowledge base reporting the standard deviation for any chosen interaction distance calculated for protein three-dimensional structures. Otherwise, it is the norm to display all bond distances for any structure at any diffraction resolution to a fixed precision, and even more remarkably usually to two decimal places, irrespective of the actual precision. The considerable variation in the *B* factors of the atoms forming chemical bonds is not recognized either, which can now readily be performed. The delay in implementing such a precision basis for structural representations has been hampered by the lack of a suitable descriptor, but the DPI approach now provides this. The example shown here (Fig. 2 ▶) is that of describing salt bridges, which are a basic aspect of protein folding and stability. The formation of salt bridges in the modelling of proteins by, for example, homology modelling, or more fundamentally in protein-folding studies based on energy minimization, are informed by the database of protein structures, but their structural precision has not been harnessed thus far.

There are other areas for immediate and obvious applications of the DPI. Deacon *et al.* (1997 ▶) illustrate one such occasion, albeit rare in protein crystallography, where full-matrix inversion is possible and the protonation states of the Asp and Glu amino acids are clear from the bond-distance σs from the X-ray crystal structure analysis (see Plate 7a in Deacon *et al.*, 1997 ▶). This naturally leads to the idea that one could broaden the examples of protonation-state assessment *via* the DPI. In addition, the DPI, being expressed in terms of experimental parameters, allows one to extrapolate to the X-­ray diffraction resolution at which the precision would become good enough to resolve such protonation states or, indeed, where neutron protein crystallography should be invoked (Fisher *et al.*, 2008 ▶). Fisher *et al.* (2012 ▶) have also evaluated the case of His, which is much more challenging for X-rays compared with neutrons. A detailed description of the biological inorganic chemistry of the bound metal environment in metalloproteins can now also be made based on the DPI; thus, the strained or unstrained nature of metal–ligand interactions can properly be assessed. Geometries such as octahedral, tetrahedral or trigonal bipyramid as common cases can then also be discriminated on a firm statistical basis *via* the DPI. A further important application of the DPI would be in the display and understanding of the stability of protein–ligand complexes and/or enzyme–substrate recognition. It will therefore be of keen interest to computational chemists engaged in drug discovery seeking to make a proper statistically based assessment of the bond interaction energies between a ligand and its cognate protein or, to put it more simply, whether hydrogen bonds, for example, are likely or not. As mentioned above, in some cases the PDB entries have average DPI values that near 1 Å (Fig. 1 ▶) and the atomic position precision descriptor shows that only integer bond-distance values are appropriate rather than real numbers; great care must obviously be exercised when using these three-dimensional structures in modelling studies.

### 4.2. Example software that calculates the DPI for a refined protein model   

*REFMAC*5 (Murshudov *et al.*, 2011 ▶) calculates DPI coordinate errors during refinement. *SHELX*-97 calculates bond-length errors using full-matrix inversion. For the different computer programs, it is important that a differentiation between the average coordinate error (σ_*x*_) and the average bond-length error (σ_*l*_) is made. The average coordinate error is based on single-atom coordinates (labelled ‘*a*’ and ‘*b*’ in equation 4) and the average bond-length error is based on the two average coordinate errors in the direction of the bond, and as such can be assumed to be 2^1/2^ times the coordinate error for isotropic atoms,
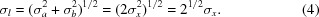



There may also be differences in the preferred usage by different software of the *R* or *R*_free_ versions of the Blow and Cruickshank equations. For instance, protein crystal structures are often (but not always) reported with the *R*_free_ reflections subset finally included in a last round of model refinement. Fortunately, the use of either in the equations gives closely similar values (Blow, 2002 ▶).

## 5. Conclusions   

In summary, SBPS has been designed and created to help to understand the nature and geometry of the ion pairs found in protein structures. The atomic positional uncertainty has been taken into account to address the error in the ionic interaction distance associated with a given salt bridge. Thus, it can be employed to shed light on the role of ion pairs in protein stability, folding, behaviour and function. Furthermore, SBPS will contribute significantly to ion-pair-related mutational studies and drug-development research. The approach used here for proper precision descriptors of salt bridges can be readily extended to metal–ligand interactions in metallo­proteins, the protonation states of ionizable amino acids and the improved understanding of protein–ligand bonding energies relevant to structure-based drug discovery.

## Supplementary Material

Screenshot of the MESPEUS view of the Mn site in concanavalin A (PDB entry 1nls). Note the truncation of the real precision of the metal ligand distances (three decimal places), which is only displayed to two decimal places.. DOI: 10.1107/S2052252513031485/mf5001sup1.pdf
