# Supplementary material for: Do we see what we should see? Describing non-covalent interactions in protein structures including precision
Source: IUCrJ. 2013 Dec 5;1(Pt 1):74–81. doi: 10.1107/S2052252513031485 (PMC4104967; doi:10.1107/S2052252513031485)
Supplement: Supplementary file 1 [file m-01-00074-sup1.pdf]

IUCrJ

Volume 1 (2014)

Supporting information for article:

Do we see what we should see? Describing non-covalent interactions in protein structures including precision

Manickam Gurusaran, Mani Shankar, Raju Nagarajan, John R. Helliwell and Kanagaraj Sekar

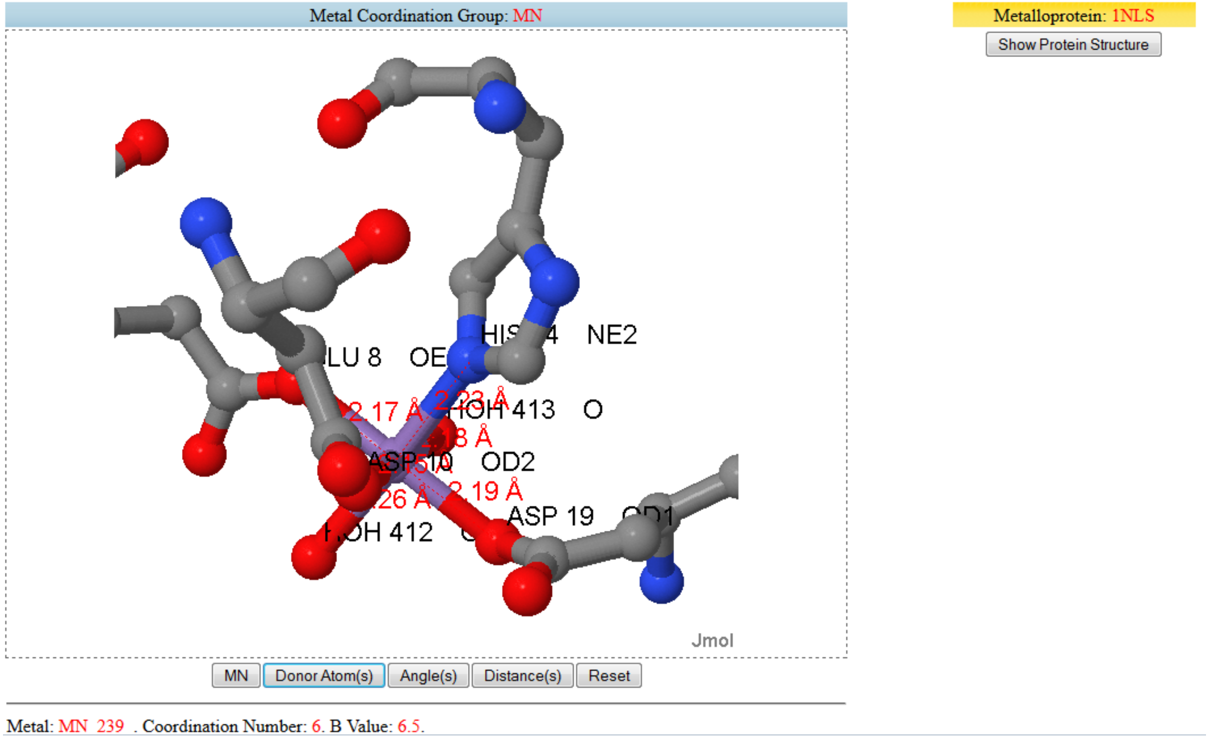

Figure S1 Screenshot of the MESPEUS view of the Mn site in concanavalin A (PDB entry 1nls). Note the truncation of the real precision of the metal ligand distances (three decimal places), which is only displayed to two decimal places.
